# The North-Eastern Hospital for Children

**Published:** 1905-05-13

**Authors:** 


### THE NORTH-EASTERN HOSPITAL FOR CHILDREN.

The Duke of Argyll has written the following letter on behalf of this hospital:—

"The North-Eastern Hospital for Children needs £9,000 to pay off a mortgage on its new building and £6,000 to make up the required income of £11,000 for the year.

"With the object of obtaining these sums, the Lord Mayor, at the suggestion of her Royal Highness Princess Henry of Battenberg, president of the Ladies' Association, has kindly consented to preside at a luncheon at the Mansion House on the 22nd inst.

"Having been associated with the hospital from its earliest years, I can testify to its deserving character, and I wish to strongly support the Lord Mayor in this effort to place it in a financially satisfactory position. The debt results from the erection and equipment of a new building, completed in 1903, by which the hospital was greatly improved in many directions, and the number of beds increased from 57 to 116, at a cost of £39,000, excluding a sum of £8,000 spent in the purchase of freehold land. All but £9,000 of this total expenditure of £47,000 has been obtained from donations and legacies, and it is this balance, which has been borrowed under a mortgage at 4 per cent., that we are now anxious to collect. At the same time it is of the utmost importance that the hospital should keep out of debt as regards its maintenance, and we therefore ask for £6,000 to ensure that this may be done in the present year.

"The fact that King Edward's Hospital Fund gives the hospital an annual subscription of £500, and has, in addition, made grants to the building fund amounting to £4,250, is sufficient evidence of satisfactory work and management.

"One of the many features of this hospital which should specially commend it to the favourable consideration of the giving public is the arrangement by which abuse of the charity is prevented and necessitous cases are assisted. For this double purpose a trained lady almoner is employed, and, by co-operation with other charitable agencies, a large amount of excellent work is done without danger of overlapping or pauperisation.

"Much more might be said in favour of so beneficent an institution, situated in the midst of some of London's poorest districts, with 116 beds in constant use and a total of over 1,400 out-patients weekly; but I must not trench too much upon your kindness, and will only add that cheques made payable to the secretary, Mr. T. Glenton-Kerr, and crossed to Barclay's, Lombard Street, may be sent to the Lord Mayor at the Mansion House or to the secretary at the hospital, Hackney Road, Bethnal Green."

